# Acute response to the October 7th hostage release: rapid development and evaluation of the novel *ReSPOND* protocol implementation within a children’s hospital

**DOI:** 10.1186/s13034-024-00767-3

**Published:** 2024-06-20

**Authors:** Naama de la Fontaine, Tamar Silberg, Jörg M. Fegert, Shlomit Tsafrir, Hana Weisman, Noa Rubin, Moshe Ashkenazi, Nitsa Nacasch, Michael L. Polliack, Wendy Chen, Meirav Herman-Raz, Ronit Wachsberg-Lachmanovich, Liat Pessach-Gelblum, Amitai Ziv, Anat Moshkovitz, Noya Shilo, Yael Frenkel-Nir, Doron Gothelf, Itai M. Pessach

**Affiliations:** 1 The Edmond and Lily Safra Children’s Hospital, Sheba Medical Center, Derech Sheba 2, Ramat Gan, 5262656 Israel; 2 Child Study Center, Yale School of Medicine, New Haven, CT USA; 3 Department of Psychology, Bar-Ilan University, Ramat-Gan, Israel; 4 Department for Child and Adolescent Psychiatry and Psychotherapy, University Medical Center, Competence Domain Mental Health Prevention, Ulm, Germany; 5 The Faculty of Medical & Health Sciences, Tel-Aviv University, Tel Aviv, Israel; 6 Sheba Medical Center, Ramat Gan, Israel; 7 Department of Social Services, Sheba Medical Center, Ramat Gan, Israel; 8 MSR-Israel Center for Medical Simulation, Sheba Medical Center, Ramat Gan, Israel; 9 Sagol School of Neuroscience, The Faculty of Medical & Health Sciences, Tel Aviv University, Tel Aviv, Israel

**Keywords:** Trauma, Acute response, Hostages, Child abduction, Hospital disaster response, Acute trauma protocol, Multilayered trauma, Childhood trauma, Parent trauma

## Abstract

**Background:**

The decision to allocate hospitals for the initial reception of hostages abducted on the October 7th Hamas attack introduced an array of unprecedented challenges. These challenges stemmed from a paucity of existing literature and protocols, lack of information regarding captivity conditions, and variability in hostage characteristics and circumstances.

**Objective:**

To describe the rapid development, implementation and evaluation of the *Hostage-ReSPOND* protocol, a comprehensive trauma-informed procedure for the care of hostages, including young children, their caregivers and families, immediately following their release from prolonged captivity.

**Methods:**

A multidisciplinary expert focus group conducted a comprehensive literature review to develop the ReSPOND protocol, consisting of: *Re*adiness of teams via multifaceted trainings, utilizing live simulations and video debriefings; *S*pecialized professional teams experienced in providing holistic trauma-informed care; *P*ersonalized care tailored to individualized and developmentally-informed needs; *O*ptimal safety rooted in creating a secure environment and trauma-informed response to young children, adolescents, caregivers and families; and *N*avigating *D*ischarge, through coordination with community-based care systems.

**Results:**

A designated facility at the Children’s hospital was carefully prepared for receiving 29 hostages, aged 3.9–80 years, 28% under the age of 18. Implementation of the *ReSPOND* protocol, which prioritized holistic psychosocial interventions above urgent medical care, proved feasible and effective in managing the diverse and complex needs of returnees as per provider report. Finally, systemic assessment of returnee’s immediate and long-term mental health needs proved highly challenging.

**Conclusions:**

There is currently no literature addressing the response to released hostages, especially those involving infants, young children and families within a children’s hospital facility. This study has the potential to fill a crucial gap in knowledge by introducing a novel protocol which could offer valuable insights for public health organizations tasked with providing acute care to diverse individuals and families experiencing extreme, multi-layered mass traumatization.

**Clinical impact statement:** This evaluation of an unprecedented protocol, developed in emergency response to the acute care of civilian hostages vastly differing in age and captivity conditions, offers insight into the unique needs of returnees affected by multi-layered trauma along with specific recommendations for large-scale response within a public health setting.

The current paper underscores the essential role of addressing the sensitive needs of children and their caregivers held in captivity, leading to the development of the novel *ReSPOND* protocol. This protocol extends crucial support not only to young captivity survivors but also to vulnerable adults.

## Introduction

The need to enhance hospitals’ preparedness has become painfully evident following devastating mass causality incidents worldwide, including the September 11th, 2001, terrorist attack, Hurricane Katrina, and the bombing in Madrid and London [1]. Consequently, response-management protocols, underlining keyprinciples appropriate for hospitals settings, have been developed [2, 3]. The Israel Center for Medical Simulation (MSR) at the Sheba Medical Center has been collaborating with civilian and military medical authorities to implement emergency trauma-management training as a national standard [4].

Despite these efforts, substantial deficiencies in hospitals’ preparedness have been reported with regards to emergency care of children [5, 6]. In fact, hospitals that have treated victims of disaster and terrorism typically employed emergency plans that lacked specific pediatric considerations [7]. Due to unique physical, developmental, and psychosocial needs, children are particularly vulnerable during mass casualty events, thus requiring specific preparation procedures for agencies involved in their immediate and long-term care [8, 9]. Furthermore, the emotional well-being of surviving caretakers also plays a significant role, influencing the potential development of psychological disorders, including posttraumatic stress disorder (PTSD) in children [10]. This becomes especially critical in the aftermath of terrorist attacks or mass violence, as parents are frequently affected alongside their children [11], underscoring the necessity for implementing comprehensive parental guidance and family-based interventions as first aid. Such preparedness proved essential for the Children’s Hospital in Nice which, due to proximity and mass casualty training, was well equipped to provide care for both child and adult victims of the 2016 Nice terrorist attack [12].Training in the provision of effective multilevel psychosocial support has also been stressed, ranging from practical support, community-based interventions, connection between different institutions, Psychological First Aid and, when indicated, specialized trauma-focused evidence-based treatments [13]. In addition, hospital professionals on the front lines of mass casualty events may experience significant mental health challenges, specifically when children are involved [14]. Symptoms of anxiety, depression, and even suicidal thoughts, as well as PTSD, are common, with healthcare workers particularly affected by caring for child victims and their families [15]. Taken together, hospital preparedness is essential for delivering effective emergency responses across multiple levels to victims of terrorism and mass casualty events involving a wide age range.

Given these shortcomings, existing protocols did not correspond specifically to the needs faced by hospitals allocated to receive and care for hostages abducted on the October 7th, 2023, Hamas terrorist attack. This paper aims to outline the shared principles guiding the Safra Children’s Hospital, at the Sheba Medical Center (a large tertiary hospital at the center of Israel) in its treatment of released Israeli hostages held in captivity for over 50 days. It also examines divergent aspects that prompted the creation and application of an innovative protocol for the immediate reception and care of both child and adult hostages within a Children’s Hospital environment.

## The unprecedented nature of the October 7th terrorist attack and abductions

The October 7th, 2023, Hamas terrorist attack on Israel involved the violent slaying of over 1,300 individuals as well as the brutal wounding of over 3,400. This attack has been regarded as unprecedented for several reasons. Firstly, it is the most extensive attack against civilians to have occurred in Israel since its inception in 1948 [16]. Transpiring on the backdrop of a political ceasefire, the attack astounded civilians, military, and government officials alike [17]. The extreme violence, including the undifferentiated massacre, torturing, burning, beheading, maiming and sexual violence committed towards individuals of all ages, nationalities and religions, often in the presence of their loved ones, is unparalleled [18]. In addition, the abduction of over 240 hostages, including 33 minors, and 40 individuals of foreign nationalities, from Israel into Gaza [19] led to an international crisis. Highly vulnerable populations were not excluded, and hostages as young as 9-months old, separated from their caregivers, elderly, as old as 86, chronically ill and disabled civilians were abducted. Consequently, grave concern for the wellbeing of injured hostages, and hostages with medical conditions, has been repeatedly expressed by health and mental-health professionals [16]. Lastly, the significant variability, in both the conditions of captivity and exposure to multi-layered violence [20], render this attack unlike any other.

## A paucity of literature

Abduction typically refers to the act of taking someone away against their will, often by compulsion or force [21]. It may also involve the sudden separation from loved ones and caregivers, acute threat to one’s life and wellbeing, and exposure to significant violence, directly and/or indirectly [22]. For children, witnessing the harm caused to a close family member, particularly a primary caregiver, is considered a traumatic exposure, and thus, the abduction of a family member, along with the experiences described above, all meet Criteria A for potentially traumatic events associated with PTSD [23]. Moreover, unlike many other traumatic events, the extreme experiences of captivity are recurrent, deliberate, often prolonged and of an interpersonal nature [24]. Undoubtedly, captivity is considered one of the most severe human-inflicted traumatic experiences [25]. Thus, significant risk of traumatization and psychosocial symptoms exist for civilians abducted in the context of armed-conflict [22], a risk compounded for abducted children [22]. However, research describing hostage reactions, particularly those involving young children, is scarce, as are studies informing evidence-based response procedures or protocols [26].

Among reports of adult hostages’ immediate reactions to captivity, denial of the danger, “frozen fright”, resulting in emotional paralysis, and “psychological infantilism”, marked by regressed behavior and reliance on captors are described [27]. Additional anxiety-related reactions, evident in the first 4 weeks post release included insomnia, fear, phobia, and tenseness. Long term reactions, evidenced 1–3 years post captivity, included irritability, increased lability, preoccupation with the experience, and feeling misunderstood [28]. Socially, hostages may withdraw, exhibit irritability, and develop a mistrustful attitude. Extended captivity also heightens the risk of learned helplessness [29].

Given that child abduction in the context of political conflict has become a recognized and growing global humanitarian problem [22], the lack of comprehensive data is concerning. In 2022, the United Nations identified 3985 cases of child abduction in the context of armed conflict. Among these cases is the abduction of over 2000 women and children by Boko Haram, an Islamic organization operating in North Nigeria. Terribly, 30 of the 276 Chibok girls abducted from their school dorms, reported experiencing physical, sexual and psychological abuse as well as torture [30]. Among the escaped Chibok girls, fear of re-abduction and sleep difficulties were reported [30, 31]. Psychological reactions, including PTSD, characterized specifically by intense shame, mortification, denial and repression of symptoms, were also reported among the 27 children who survived the Chowchilla bus abduction in 1976. A recent review of the impact of abduction on children and adolescents worldwide [22] details an array of significant reactions documented among hostages including PTSD, depression, appetitive aggression (e.g., pleasure in committing violent acts), physical symptoms (e.g., dermatological complaints, muscle and abdominal aches, malnutrition, dysregulation to menstrual cycle), as well as behavior and cognitive difficulties. These reactions are associated with both pre-existing individual and familial circumstances as well as with abduction related exposure. Taken together, hostages, and particularly children, abducted on October 7th are at great risk for multiple and extreme traumatization as well as trauma-related symptoms, requiring interventions appropriate for both immediate and long-term reactions.

Nearly two months after the October 7th attack, 105 civilian hostages (81 Israelis, 23 Thai nationals, and one Filipino), including infants as young as three years old, mothers, young females, and elderly women up to 85 years old, abducted from their homes at the Kibbutzim in the Gaza envelope and from the Supernova peace festival, were released through negotiation. In a major effort to mitigate harm, the preparation for their return was given high national priority. Subsequently, the local healthcare community was confronted with the need to respond to an unprecedented mass-victim event marked by extraordinary complexity and variability. Lacking any certainty regarding their physical and mental conditions, hospitals were assigned as the first site to receive hostages. To meet this task, and in line with the Ministry of Health recommendations, the Safra Children’s Hospital, located within the Sheba Medical Center in Israel, was among the first in the world to develop a multi-layered comprehensive protocol for the reception and acute care of released child and adult hostages.

This paper aims to describe the expedited process of developing the Hospital's initial response by addressing both anticipated and observed needs of returnees. Specifically, we aim to (a) describe the process by which we created the *Hostage - ReSPOND protocol* that stands for: **Re**adiness, **S**pecialized teams, **P**ersonalized care, **O**ptimal safety and **N**avigating **D**ischarge to be implemented with diverse civilian hostages, ranging considerably in age and circumstances, during the initial 24–48 hours of their release from captivity; (b) review the implementation of the *Hostage*-*ReSPOND* protocol within the Sheba Medical Center, Safra Children’s Hospital, and (c) assess the feasibility and usefulness of the protocol in swiftly addressing the varied needs of hostages and organizational requirements, by examining strengths, weaknesses, and insights gained from the novel protocol.

## Methods

The study was approved by the Institutional Review Board of the Sheba Medical Center- Tel Hashomer (0947-23-SMC) Israel, and conducted according to the Declaration of Helsinki.

First, an interdisciplinary senior staff focus group, comprised of adult and pediatric physicians, psychiatrists, psychologists, social workers, nurses and Public Relations personnel was organized. Selection of focus group members was based on level of clinical experience and diversity, with the intention of incorporating varied areas of clinical expertise and considerable experience with child and adult trauma. The focus group met once or twice weekly to define and facilitate the implementation of protocol guidelines. Discussions were structured around a set of carefully predetermined questions and were moderated by the Director of the Children’s Hospital (I.M.P.).

Second, a rapid review of the literature was conducted in order to assess existing literature on hostage and captivity situations, specifically those involving young children and child-caregiver dyads. The search included electronic databases, such as MEDLINE, EMBASE, and Cochrane Central Register of Controlled Trials. Studies were included if they were published between 1970 and October 2023 and written in English. Quantitative, qualitative, case reports, reviews and mixed-method studies were included to consider different aspects of captivity within the civilian population. Literature review keywords included: abduct*, captivity*, hostage*, kidnap*, child kidnap*, parent abduction*. Next, the search strategy was expanded to include the keywords: “terrorism”, “war”, “terror*”, “terrorist attack”, as well as “armed conflict”, “acute trauma response*” and “post traumatic response. However, due to the unique circumstances surrounding the situation under study, as well as the relatively short timeframe in which the review was conducted, we opted for a non-systematic approach to gather and analyze the available information. This decision was made to ensure timely access to relevant data and insights in a rapidly evolving context. In addition, local expert guidelines (such as the national council for the child, the Ministry of Health, Israel Defense Force: IDF) informed our approach. Further information was also gathered through personal communication with international experts in the field of hostage situations, child and adult trauma and disaster response.

Based on these cumulative findings, the focus group arrived at a consensus document which details key principles and practical guidelines for the initial reception and care of hostages, (from here on referred to as “returnees”), across the age range. Considerable attention was given to the unique needs of pediatric hostages, parent-child dyads, and families. In addition, guidelines encouraged careful consideration of individual characteristics and circumstances of hostages (i.e., preexisting psychosocial risk and protective factors), as well as the variance in the traumatic exposure during the terror-attack and while in captivity (highlights from the document are provided in Table 1). Guidelines were reviewed and reaffirmed by the Israeli Child National Council. Leaders of clinical sectors reviewed these guidelines with all staff members, and strategies, specific to each sector, were further developed.

**Table 1:** *ReSPOND* guidelines for the medical center clinical staff provided by the focus group

Topic	Key principles		Practical guidelines	
Readiness	1. Customized trauma-informed acute training 2. Utilization of live and videotaped simulation-based trainings 3. Acknowledgment of shared traumatic experience for team members 4. Preparing facility to regulate arousal and enhance sense of security		• Identify common and individualized trauma-related reactions • Integrate insights based on dilemmas presented in trainings • Provide staff education, supervision, and support to recognize and respond to effects of complex multilayered trauma • Monitor colleagues’ responses and needs • Prepare facility to reduce overstimulation (e.g., lighting, odor, noise)	
Specialized teams	1. Collaboration within multidisciplinary teams 2. Synchronization of organized team response to mental and physical needs 3. Social workers and psychologists as core responders and case-managers 4. Consultation with senior supervisors		• Conduct brief periodic multidisciplinary and supervision team meetings • Follow protocol intervention steps (e.g., prioritize attention to mental health status before non-urgent medical procedure) • Psychologists assigned to each released individual or child-parent dyad • Social workers assigned to accompany returnee’s families	
Personalized and professional acute care	1. Provision of individually tailored trauma-informed response 2. Evaluation of pre-existing risk factors 3. Involvement of family members in preparation for returnees and throughout admission		• Gather psychosocial and medical information beforehand • Ensure age-appropriate, individually-tailored setting • Utilize trauma and developmentally informed language • Collect from family members personal items (e.g., transitional objects) for children • Address family members’ needs and balance those with returnees' needs	
Optimal safety	1. Reduction of initial distress and regulation of arousal 2. Provision of individually tailored basic physical and mental needs 3. Response to acute trauma reactions 4. Breaking bad news 5. Balancing between personal sense of security and national level security needs		• Enable privacy as best possible, consider minimal media intrusions and exposure • Respect relationships formed among returnees during captivity • Use clear, transparent and age-appropriate communication to address trauma reactions • Obtain verbal consent prior to providing physical assistance • Enhance caregiver stabilization and active involvement • Prepare for the delivery and reception of bad news • Advocate for returnees’ needs and rights	
Navigating discharge	1. Mediation of needs associated with hospitalization, release timing and procedure 2. Recognition of stress reactions associated with discharge and future adjustment 3. Collaboration with multidisciplinary team on comprehensive discharge plan 4. Communication with community-based health-care systems		• Conduct pre-discharge multidisciplinary meeting • Conduct discharge session with returnees and family members tailored to individualized needs (i.e., parent guidance, adolescent and child “goodbye” sessions) • Provide psychoeducation, including written materials, regarding common long-term trauma reactions and parent guidelines • Facilitate ad-hoc connection with community-based long-term care systems • Manage multilayered needs associated displaced returnees and their support network	

These guidelines were further developed into the elaborated *ReSPOND* protocol as outlined below:

### Readiness

A specialized, trauma-focused training was developed for the multidisciplinary team, comprised of (a) lectures on acute stress and posttraumatic stress reactions across the lifespan, emphasizing diverse and compounded traumatic exposures (e.g., physical and sexual assault, parent-child separation, food and stimuli deprivation, traumatic grief and death notification); (b) live simulation-based trainings and video-based debriefing sessions conducted with professional actors and members of the medical and psychosocial teams at MSR. Studies suggest that active engagement in the learning process within a meaningful or relevant context is crucial for fostering adaptive work-related expertise, a goal that can be achieved through live video simulations [32]. In the current hostage situation, training simulation scenarios were developed by senior trauma experts who composed scripts anticipating returnee’s physiological, psychiatric, and emotional conditions, as well as scenarios expected to unfold throughout their hospital stay. Scripts were created for both adult and adolescent returnees, as well as for mother-infant dyads, highlighting the common and divergent factors in caring for returnees across the age range. Scripts incorporated pre-defined tasks, including pre-reunification family support, returnee admission and escort to unit, family reunification, death notification, developmentally-informed interventions, medical exams, and hospital discharge procedures.

Simulation scenarios were developed to raise awareness and develop strategies for effective coping with potential challenges, inform specific language, and holistic, developmentally-informed methods of care. Special attention was given to the unique needs and care of pediatric returnees (e.g., the importance of a familiar and self-regulated caregiver present at all times and strategies for facilitating positive parental involvement), as well as the importance of constantly monitoring and supporting returnee family members. Live simulation sessions were followed by video-based debriefing, focus-group discussions and group supervision by the senior expert team, refining system-level coordination and elucidating key guidelines for privacy protection, media management, chain of command, decision-making, and task prioritization. Identification of various dilemmas presented by professional actors (such as separation anxiety, refusal of medical exam, dissociation and disorientation during simulations) led to protocol refinement and promoted teamwork and skill mastery.

The extreme hostage situation, intertwined with the tragic events of October 7th and its aftermath, clearly challenged healthcare teams as well. The extreme situation necessitated interdisciplinary providers to deliver acute care that addressed multiple trauma layers within an ongoing national security threat. A shared traumatic reality has been identified in previous research as a significant risk factor for healthcare providers’ personal and professional well-being [33]. Thus, the preparedness of professional teams for such an extreme traumatic event highlighted the need for incorporating trauma-informed principles to prevent secondary traumatic stress. This included shared sessions with senior trauma psychiatrists, relaxation techniques, enabling open communication and sharing of distressing emotions, and a designated space with refreshments for rest and team bonding.

### Specialized teams

Past hospital responses to mass casualty incidences highlight the importance of prioritizing the organization of human response over physical supplies [34]. Accordingly, considerable efforts were devoted to the formation of well-rounded specialized teams. The teams dedicated to receiving and caring for the returnees consisted of experienced adult- and child-focused multidisciplinary professionals, from the following sectors: medicine, social work, psychology, forensics, gynecology and nursing. As a general standard and principle, the Sheba Medical Center ensures all aspects of diversity are represented as best possible.

Psychologists and social workers functioned at the core, with the former assigned to each released individual or child-parent dyad, and the latter assigned to accompany returnee’s families who awaited the release of their loved ones within the designated hospital facility. Each pair was reinforced by a team supervisor, a senior psychiatrist, psychologist, or social worker, present on site and available for consultations as needed. In addition, forensic specialists were available on site and called upon when a forensic exam was indicated. Nurses and supportive staff were positioned throughout the facility. Director and Deputy Director of the Safra Children’s Hospital oversaw the entire operation and were present at the facility from reception to discharge.

**Fig. 1:**
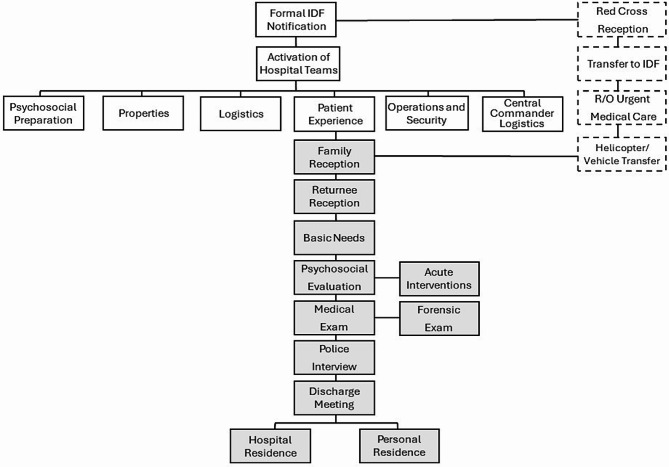
Protocol for multi-disciplinary reception and care procedures of hostages admitted to the Safra Children’s Hospital. Dashed lines indicate returnees’ release process outside hospital ward; Straight lines represent hospital arrangements for the reception of returnees; Grey rectangles signify the process within the hospital facility following returnees’ admission. *IDF-Israeli Defense Forces

A primary task of the professional teams was optimizing and streamlining the transition process from the Gaza border and throughout returnees’ stay (see Fig. 1).

Upon receiving military notification with names of hostages expected to be released, on-call unit leaders would be contacted and a multidisciplinary team, encompassing psychosocial preparation, properties, logistics, patient experience (e.g., basic necessities, hygiene, food supply, cleaning crew), operations and security (i.e., media control, spatial management, security of premises), and central commander logistics were to be activated.

A dedicated and integrated authority was established by the state, tasked with collecting information from various parties (e.g., Israeli Defense Force: IDF, Ministry of Social Services, Ministry of Health, Hostages and Missing Family Forum). This authority created a background file for each returnee and forwarded it to the hospital staff as soon as their return was certain. Psychosocial and medical teams would then receive background information related to returnees’ age, family structure, significant losses, traumatic exposure and any information available pertaining to preexisting medical or psychosocial conditions. Social workers were tasked with receiving pre-designated family members of returnees, providing support to awaiting families and counseling them in preparation for their reunion with returnees, while psychologists attended to the returnees at the hospital entrance and guided them through the initial step of family reunification.

To ensure privacy, the entrance of the Safra Children’s Hospital was blocked off from the media and unverified visitors or personnel. Entrance to the facility was permitted only to identified staff members, which received a specific name tag and hand-band. Family reunification was highly prioritized and therefore, returnees would be immediately accompanied to meet their families in the privacy of their designated rooms. Next, provision of basic needs (e.g., preferred meals, shower, change of clothes, hygiene items), would be attended to, followed by an evaluation of mental state and needs, introduction to medical personnel and invitation to conduct medical exam. If evidence of sexual or physical abuse is detected or disclosed by the returnee, appropriate professionals would be consulted to determine the feasibility of collecting further evidence without exacerbating distress. If needed, forensic exams would be offered as well. Finally, interviews with a designated, pre-trained security intelligence officer would be offered to all returnees above the age of 14, to allow for the gathering of crucial information pertaining to the fate of hostages who were still held in captivity. Next, in agreement with returnees and their families, discharge meetings would be conducted during which a discharge plan would be made.

### Personalized-care

A vital goal of acute interventions is the early identification of trauma related reactions and the delivery of preventive interventions that could reduce the risk for acute stress disorder and PTSD [35]. To this end, and in line with the acute trauma-informed approach, screening for both acute and post-acute traumatic stress responses was to be performed [36]. Standardized measures that would rely upon returnees’ self-report were deemed inappropriate due to the sensitive, and highly unpredictable, conditions of returnees. Instead, screening relied on clinical observations, both documented in returnees’ medical files and through clinician-rated responses to modified standardized measures as described below:

A clinician- rated child symptom checklist was developed by the authors and sent via a secure electronic link to all clinicians. The checklist was based on the Trauma Symptom Checklist for Young Children (TSCYC) [37], a 90 item scale comprised of eight-factors for young children, and the Trauma Symptom Checklist Short Form (TSCC- SF) [38], a six-factor, 29-item scale for children and adolescents. Both measures pertain to children’s trauma- and abuse-related symptomatology as assessed by caretaker report, and were found to have well established psychometric properties [39].

Caregiver symptoms were assessed using the Adult National Stressful Events Survey Acute Stress Disorder Short Scale [NSESSS] [40, 41], a 7-item measure evaluating the severity of acute stress disorder symptoms in individuals age 18 and older following an extremely stressful event or experience. The scores for both child and adult scaled items were dichotomized to ‘present’ or ‘absent’. Findings pertaining to returnee physical symptoms were collected from medical files.

### Optimal safety

**Fig. 2:**
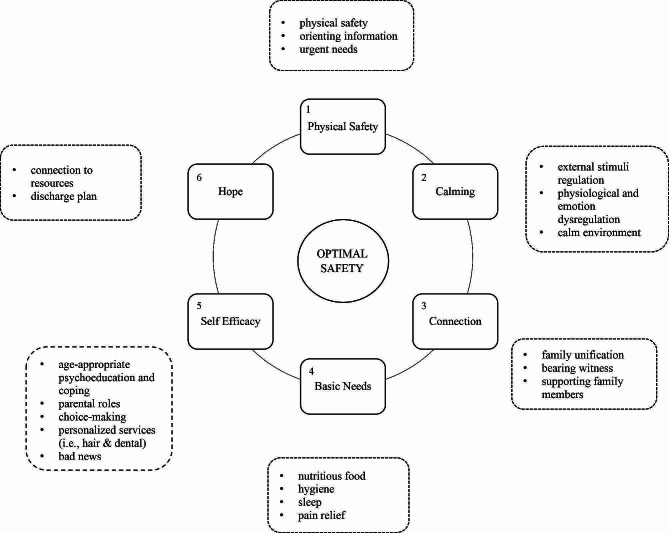
Basic components of Optimal Safety, including, specification of primary action items depicted in boxes

A core principle in providing psychological first-aid response to acute trauma survivors consists of regaining a sense of control and safety [42]. This is usually attained by providing survivors with information and space to express their concerns while ensuring that their needs and voices are heard [43]. To this end, the *Optimal safety* component focuses on well-recognized reciprocal elements comprising Psychological First Aid [44], with particular attention given to developmentally-informed needs. These elements consist of (1) physical safety, (2) calming, (3) promoting connectedness, (4) attending to returnees’ basic needs, (5) promoting self-efficacy, and (6) instilling hope, gradually implemented and individually tailored to ensure optimal safety, as delineated in Fig. 2.

#### (1) Physical Safety  

Physical Safety refers to the importance of providing food, protection, and connectedness [44, 45]. All returnees would be primarily received by the Red Cross and transferred to the IDF special reception teams (see Fig. 1 for Release Process). IDF medical personnel would assess whether urgent medical care is indicated, in which case returnees would be transported to the nearest medical center. If urgent medical care is ruled out, returnees would be provided with basic elementary needs, including: change of clothes, water and snacks. When possible, IDF personnel would engage children and parents through play and provide orienting information, including a verbal account of their intended next steps (i.e., transportation via helicopter or vehicle to the Safra Children’s Hospital, where their families await).

#### (2) Calming

Given prevalent neurophysiological dysregulation following traumatic exposure [46, 47], staff were trained to maintain a calm, compassionate presence, limiting demands placed on returnees and providing accurate information [48]. Particular attention would be devoted to supporting caregivers as studies demonstrate the vital co-regulatory role of caregivers for young children [49], as well as the crucial impact of parent–adolescent interactions in the aftermath of highly stressful events [50]. Therefore, all caregivers would be provided with professional support aimed at self-stabilization and parental guidance.

Considerable attention was also devoted to returnees’ hospital environment. After careful consideration, a dedicated facility within the Safra Children’s Hospital was chosen. This unit was tailored to meet the needs of children, adolescents, and families. It features child-friendly décor, private rooms, and communal areas to provide flexibility in privacy and social interaction, shielding returnees from unwanted exposure to visitors or experiences shared by fellow returnees. Dim lighting would be maintained within the facility to counter potential effects of light deprivation during captivity. Staff were trained to speak softly and to minimize their presence, to allow for returnees’ privacy and comfort. Steps were taken to protect returnees from visitors, media, and unwanted external stimuli. Furthermore, the enrolment of female staff members was preferred to mitigate power dynamics and establish a sense of safety, especially as traumatic experiences were primarily inflicted upon hostages by male perpetrators and some evidence exists indicating preferences for female providers, particularly pertinent to disclosure of sexual abuse [51].

#### (3) Connection

Human connection enhances a sense of continuity in face of disruption and trauma, provides models of resiliency which could bolster survivors’ own sense of competence, and increases their access to tangible and non-tangible resources [52]. Accordingly, family reunification has repeatedly been cited as a priority in disaster and mass trauma response, especially for children and adolescents for whom the stress resulting from delayed family reunification has been associated with significant and prolonged negative effects [53, 54]. Appropriately the World Health Organization (WHO) advocates for family reunification as an essential component of promoting mental health in post-disaster response [55]. Connection would therefore be facilitated in several ways. For instance, core teams would meet returnees at the entrance of the Children’s Hospital to establish a personal connection, and immediately escort them to their closest family members, where they would reunite in a private setting before gradually connecting with additional family members.

#### (4) Basic needs

 Addressing children's and families’ basic needs is crucial for restoring stability and promoting a sense of control following traumatic exposure [42]. Once physical safety is ensured and returnees reunite with their families, food, shower facilities, and clothing would be offered, with specific attention given to providing well-fitting and age-appropriate clothing, as oversized or ill fitted clothing could serve as trauma reminders [56]. Potential developmental regression was taken into account with regards to all returnees, therefore, different needs would be prioritized based upon age, ongoing clinical observation, and communication with returnees and their family members. For example, earlier access to their peer group would be facilitated for adolescents, while younger children would be given toys and other comforting items. Adults and care givers would be prioritized with regards to access to information.

#### (5) Self-efficacy

Adult returnees would be encouraged to make personal choices regarding their hospital stay duration, environment, media exposure, and medical/psychological care. Parents would be encouraged to facilitate these decisions for their children, in a developmentally appropriate manner, with the aim of empowering and re-establishing parental roles. In addition, services such as dental care, haircuts, and cosmetic care would be offered upon request, as these are linked to personal identity and self-efficacy [57]. Retrieving personal items and allowing personal choices, including hairstyles, are important aspects of survivors’ identity reclamation process [58].

To further enhance self-efficacy, self-regulation and independence, evidence-based trauma-informed interventions were included in the protocol. Soon after their arrival to the hospital, all families and returnees would be informed of the expected course of events to establish predictability. Psychoeducation on expected psychological, physiological, emotional, and behavioral reactions, along with immediate and long-term response recommendations, would also be provided. The team would prioritize validating and supporting family members’ reactions, concerns, and needs. They would provide training in basic relaxation and cognitive coping skills to allow regulation of arousal, enhance resilience and manage various stress responses. Returnees with children would be offered parent guidance, including psychoeducation on developmentally informed traumatic stress reactions in children and the crucial role of primary caregivers in identifying these reactions and intervening early by re-establishing predictability, safety and open developmentally-informed communication. This information would be shared privately with parents or, when feasible, in dyad sessions, facilitating deeper understanding of anticipated stress and trauma-related responses and recommended trauma-informed practices.

As family members were expected to provide devastating news regarding their community, other family members or friends that were either murdered or are still held in captivity, notification protocols were to be followed, including SPIKES (Setting, Perception, Invitation, Knowledge and Empathy), often used by medical professionals [59]. Special care would be taken when delivering bad news to children and adolescents. Teams would then offer guidance to caregivers and, when requested, deliver bad news alongside family members. Whenever possible, psychoeducation on traumatic grief responses would be provided to adults, parents, and children, with specific developmental needs in mind.

#### (6) Hope

A central objective of terrorism is fostering hopelessness [60]. One aspect of hopelessness is a sense of a foreshortened future, which is well-recognized as a prominent posttraumatic symptom [61]. Following disaster, adolescents have conveyed a decrease in hopeful thinking [62]. Therefore, a vital component of Psychological First Aid is facilitating hope. Survivors, who often perceive challenges stemming from mass trauma as one unsolvable problem, could greatly benefit from enhancing problem-solving strategies. Not only does this help prioritize the overwhelming bombardment of practical tasks that they face, but it could also enhance their sense of control and instill hope [63]. This would be achieved by the provision of developmentally informed psychoeducation, connection to psychosocial, communal and medical resources, and delineation of a detailed discharge plan. For parents, these interventions could reinstate their sense of control and authority, subsequently reassuring their children and instilling hope in them as well. For adolescents, who rely heavily on their parents and peers, special attention was placed on gradually re-connecting them to their social resources by reassuring them that their network has been eagerly awaiting their safe return.

### Navigating discharge

Recognizing potential future deterioration, we followed post disaster recommendations and intended to coordinate care continuity through careful planning of pragmatic manageable steps [64, 65]. Upon hospital discharge, family members and parents were to be provided with brochures and handouts including important information regarding developmentally informed expected reactions, review of principles for coping with such reactions, and information necessary in case they wish to reach out to Sheba staff members in the future. In addition, connection to community-based resources was a major priority led primarily by the psychosocial teams. Follow up was to be conducted a week and two weeks after discharge, by telephone and in some cases in person, as directed by the Ministry of Health. This was to ensure that needs and wants were met once the returnees were in the community.

Assessment of the *ReSPOND* protocol implementation and feasibility was obtained via multi-level supervision conducted by highly experienced adult- and child-trauma as well as medicine experts. This team oversaw protocol development and implementation beginning with training; supervisors participated in live simulations, observed trainees undergo live and video-based simulations, and offered immediate on-site feedback, modeling and guidance. Therefore, feasibility would be assessed based upon supervisors’ feedback pertaining to the *ReSPOND* protocol implementation.

## Results

Twenty-nine hostages, including 8 children (mean age 11.47 *±* 4.37, range 3.75-17.0) and 21 adults (mean age 52.43 *±* 19.65, range 18–84) were admitted to the Sheba Medical Center, in three pulses, from November 25th to November 30th, 2023 (see Table 2 for returnees’ demographic data).

**Table 2:** Returnee demographic data (*N* = 29)

Demographic Data	*N*	%
*Sex*		
Females	26	89
*Age*		
Under 18	8	28
Over 18	21	72
*Place of residence*		
Gaza envelope	16	55.2
Outside of Gaza envelope	13	44.8
*Place of abduction*		
Primary residence	15	51.7
Friend/relative residence	11	37.9
Nova music festival	3	10.3
Marital status*		
Single	4	19
Married	10	47.6
Divorced	2	9.5
Widowed**	2	9.5
Committed relationship	3	14.3

Notes: * Of the 21 returnees above age 18; ** Prior to the October 7th attack.

Returnees stayed within the hospital for 24 to 72 hours post release, with the exception of one minor whose caregiver’s release was unexpectedly denied by Hamas, resulting in profound uncertainty as to her fate. The child remained in the hospital for a week until her caregiver’s release.

### Readiness of Professional teams

Professional teams were confronted with returnee’s experience of extreme multilayered trauma, as returnees not only struggled with their own experiences of captivity, but also learned about the tragic loss of family and community members as well the destruction of their homes. The combination of recounted grief responses and the distressing descriptions of traumatic exposure and conditions of captivity, characterized by abusive and aggression, deeply impacted health professionals. In the aftermath of providing acute response to returnees, several team members sought professional emotional support. Senior psychiatrists and psychosocial staff were readily available to provide support. Supervisors initiated personal check-ins with their teams, and several attempts were made to provide structured group-based support.

### Personalized care

Although none of the hostages required emergency medical interventions upon arriving at the hospital, signs of captivity were evident. These post-release symptoms included: signs of starvation and lack of adequate feeding (weight loss, loss of muscle mass and significant nutritional deficits), deficient hygiene (e.g., lice, scabies), mistreated physical injuries, severe medical neglect, and medical negligence of pre-existing medical conditions (e.g., chronic diseases such as hypothyroidism, diabetes, hypertension and heart failure) as well as morbidities that developed during captivity (including, but not limited to, neuropathies, heart failure, thrombosis, skin disorders, malnutrition). In addition, signs of physical and sexual abuse were evident in some returnees (e.g., body markings). A summary of returnees’ physical conditions, as reported in their medical records, is presented in Table 3. A detailed description of the physical and medical implications of captivity will be reported elsewhere.

**Table 3:** A summary of returnees' physical and medical manifestations

Physical conditions			
Reported conditions	*N*	Frequency	%
Pre-existing medical condition	29	15	52
Complications of pre-existing medical condition	29	10	34
Physical injury during abduction	29	25	86
Significant weight loss (> 6% of baseline weight)	29	18	62
New significant morbidities that developed in captivity	29	8	28
Reported Physical abuse	29	21	72

In terms of psychological responses, returnees’ presentations were diverse and included avoidance, anxiety, sadness, anger, and signs of regressive behaviors (e.g., clinginess, and lack of assertiveness in children and adults). Hyper-arousal reactions were highly evident in both children and adults, all of whom refrained from sleeping in the hours following their hospital admission (see Tables 4 and 5 for a description of child and caregiver responses, respectively). Other responses, revealing the immediate impact of captivity, were also evident; for example, young children who conveyed being ordered to “*USKUT*” (“Be Quiet” in Arabic) and communicated entirely in whispers, despite repeated prompts to speak freely.

**Table 4:** Clinician-rated child acute symptoms as assessed by modified versions of the TSCC and TSYC

Child symptoms (*N* = 8)			
**Symptom cluster**	**Symptom**	*N*	**%**
**Intrusive symptoms**			
	Upset by memories	8	100.0
	Cries when remembers	7	87.5
	Panics when remembers	7	87.5
	Re-experiences event	5	62.5
**Dissociative symptoms**			
	Stares	6	75.0
	Lacks concentration, "in his/her own world"	6	75.0
**Arousal symptoms**			
	Startles	8	100.0
	Easily frightened	8	100.0
	Checks for danger	6	75.0
**Avoidance symptoms**			
	Avoids talking about experience	8	100.0
	Avoids memories	8	100.0
	Displays no feeling	6	75.0
	Denies experience	6	75.0
	Can't recall experience	4	50.0
**Anxiety symptoms**			
	Worries about safety of others	8	100.0
	Scared to be alone	7	87.5
	Scared of the dark	5	62.5
**Depressive symptoms**			
	Sad or depressed	6	75.0
	Avoids playing	4	50.0
	Avoids laughing	5	62.5
**Atypical symptoms**			
	Worries food is poisoned	3	37.5
	Wants to die	3	37.5
	Forgets his/her name	1	12.5
**Aggressive symptoms**			
	Overly aggressive	3	37.5
	Yells at others	2	25.0
**Sexual symptoms**			
	Sexual concerns	2	25.0

*TSCC* Trauma Symptom Checklist; *TSYC* Trauma Symptom Checklist for Young Children

**Table 5:** Clinician-rated caregiver symptoms

Parent symptoms (*N* = 6)		
Symptom	n	%
Flashbacks	4	66.7
Upset	6	100.0
Detached	4	66.7
Avoid	6	100.0
Alert	6	100.0
Startle	6	100.0
Nervous	4	66.7

In addition to trauma-related symptoms, some returnees also expressed positive reactions such as optimism, relief, seeming relatively oriented to their environment, and engaging freely with their family members. Similarly, several young children were able to express their needs to staff members (e.g., requesting specific foods and games). Parents typically prioritized privacy and requested assistance in keeping media representatives away. They also made requests regarding assistance in pacing and timing of their reunification with extended family members.

Regarding reactions of accompanying family members, some requested clear guidance concerning recommended responses to returnees, and for the management of their own reactions. Regardless, pertinent guidelines were provided to all family members as part of the preparation for returnee arrival, and throughout admission, as specified in Table 1. While expecting the arrival of returnees, and throughout their hospital stay, varied practical requests were often put forth to the staff by accompanying family members (e.g., requests to rearrange the lighting or designated rooms, adjust meals, and or manage the various procedures offered). Beyond practical care, these were understood as potential manifestations of family members’ distress or anxiety and were therefore accommodated by all staff (regardless of seniority, sector or specialization) with considerable flexibility and acceptance. In this manner, a well-functioning, self-sufficient and highly adaptable ecosystem was maintained within the unit.

In terms of providing individually tailored response, partial critical background information was obtained in the hours prior to receiving the list of hostages expected to be released. Hence, prior to admission, crucial details emerged (e.g., the need for prescription eyewear or medication) allowing for individually tailored preparation. Returnee rooms were arranged with extreme care, considering any existing knowledge concerning age, gender, medical needs, and personal preferences including for example, attention to returnees’ lodging arrangement in terms of their proximity to other returnees and family members. Information regarding traumatic exposure (albeit limited) which was gathered from the national authority and from family members and relatives, allowed for initial treatment planning, alerting medical teams when injuries were to be anticipated. Developmentally informed decisions were made based upon returnees’ age, such that necessities and comforting items (e.g., teddy bears, blankets, slippers, new cell phones) were distributed in accordance with returnees’ expected needs. In addition, when possible, family members were encouraged to place personalized items from home in returnees’ rooms, an anticipatory act that clearly held significant emotional meaning. Even personal pets were brought to the hospital facility when it was deemed that the child’s strong attachment to them would provide a sense of continuity, comfort and support.

### Optimal safety

All returnees received stepped care in the sense that, once the need for urgent medical care was ruled out, basic needs were prioritized, followed by psychological, legal, practical and social needs. As many returnees seemed undernourished, proper nutrition was provided. This was done in a gradual manner as to prevent Refeeding Syndrome, a potentially fatal condition, caused by giving an undernourished person too much food and fluids too quickly, leading to metabolic disorders and fluid and electrolyte imbalances [66].

As expected, a great deal of time was designated for the private reunification of returnees and their families, as they engaged in a natural process of re-acclimation [67]. Most returnees and their families accepted acute psychological interventions offered by staff, at times requesting their guidance and presence. Interventions delivered included trauma and grief related psychoeducation [68], relaxation strategies, parent guidance and support in the provision of difficult news, including death notification and notification of remaining family members in captivity. The harm caused to returnees’ family members, the circumstances of captivity and condition of returnees upon hospital admission and discharge are presented in Table 5.

Due to the extremely sensitive situation, additional roles taken by the psychosocial teams included advocacy aimed at protecting returnees’ privacy and assisting them, if they desired, through medical and special interview procedures with security intelligence officers.

### Navigating discharge

Most families expressed a desire to prolong their hospital stay beyond that expected, demonstrating effectiveness in creating a transient space for returnees. Families were given the option of complete discharge within 24–72 hours, or discharge to a hotel at the medical center facility. For those who accepted the latter, low intensity continued care was provided in the days following discharge from the unit (see Table 5). Some expressed a wish to clear out their rooms in hopes that it would be shortly occupied with additional released hostages. Upon discharge some children expressed fear of re-abduction and shared that they were told by their abductors that they will “come and find them wherever they are”. For providers, some expressed concerns with release within 24 hours, while others postulated that discharge would enhance a sense of self-efficacy and encourage returnees to take an active role in planning their next steps towards independence. Nevertheless, length of stay was typically 24–48 hours.

Efforts at multi-system discharge synchronization proved challenging as most returnees lost their homes and their communities in the October 7th massacre, and were evacuated to temporary lodgings dispersed throughout the country. Prior to discharge, multi-agency conferences were held for each returnee and family to coordinate continued care in accordance with their personalized needs and choices. Every family was provided with the option for ongoing mental health support at the Sheba Medical Center’s adult and child trauma outpatient clinics. However, apart from two young adults, all families opted not to continue care at the hospital clinics, but rather, expressed a preference for receiving mental health support closer to their current residence (see Table 3). The hospital’s social workers conducted follow-up calls to community social workers, who were appointed in advance and trained by the Ministry of Welfare and Social Affairs to deal with the sensitive returnee situation. This ensured an organized and systemic follow-up procedure across all cases.

## Discussion

In the following discussion, we present the insights gained from the transition from anticipated to actual needs, including intervention feasibility and limitations, along with the initial conclusions of the implementation of the *ReSPOND* protocol developed at the Safra Children’s’ Hospital, the Sheba Medical Center, Israel. In general, the protocol seemed feasible and was implemented as expected, with specific facilitators and barriers identified throughout the implementation process.

### Readiness

Research on simulation-based training has significantly advanced over the past 15 years, highlighting its potential benefits in academic and medical settings [69]. However, to the best of our knowledge, this is the first application of simulation-based training in preparation for both adult and child-focused responses to an unprecedented disaster situation, such as hostage release. Lessons learned from the training simulations have led to the development of a collaborative interdisciplinary approach, enabling staff members to navigate complex situations together and effectively prioritize returnees’ individualized needs. Furthermore, the use of live and videotaped simulation-based trainings has allowed staff to practice in advance unprecedented scenarios anticipated during the returnees’ hospital stay, promoting preparedness and skill mastery.

### Specialized teams

The circumstance that nearly a third of the freed hostages were children under 14 years old intensified the emotional impact of the situation on healthcare providers. A shared traumatic reality has been identified in previous research as a risk factor to healthcare providers’ personal and professional well-being, underscoring the need to provide education, supervision, and support to mitigate the possible negative effects of shared trauma [33]. Nevertheless, providers across all sectors also expressed exceptional motivation throughout, evidenced by their unwavering commitment to best meet the physical, professional and emotional demands placed on them. Given the opportunity, most expressed the wish to care for additional returnees. This could be understood in terms of compassion satisfaction, stemming from providers’ ability to connect with, and help others, in a manner that outweighs the intense stress associated with their work [70]. Thus, working with the returnees had a personal impact on many team members to differing extents, as they attended to a highly vulnerable population and implemented an unprecedented protocol during a particularly vulnerable time.

Despite the advantage of integrating multidisciplinary teams to provide holistic trauma-informed care, challenges were identified. For example, expertise in child versus adult trauma response evoked discrepancies in approaches and practices. Furthermore, despite the teams being composed of trauma-expert psychologists and social workers, with extensive experience in pediatric trauma, this first professional collaboration during a national-level catastrophe presented significant challenges. Challenges pertaining to division of labor, fluidity of care and collaboration surfaced, with little time for preparation. Undoubtedly, these factors impacted boundaries and roles, ultimately contributing to significant stress. Our findings are in line with previous studies reporting on challenges associated with the *blurring of roles* often recognized among mental-health professionals during stressful work-related situations, specifically between psychologists and social workers [71]. Such concern aligns with previous studies highlighting that confusion between roles is a challenge in teamwork dynamics, which hinders effective team functioning [72].

Lastly, despite occasional efforts to provide professional supervisory support during returnee hospital stay, the lack of structured debriefing sessions, implemented consistently across all sectors, limited the opportunity for comprehensive support, and possibly hampered staff’s ability to cope more effectively with their experiences.

### Personalized care

Diverse physical and psychological reactions were identified, which were generally more positive than anticipated. This may underscore our team’s bias towards anticipating negative reactions, potentially overlooking previously documented positive or adaptive responses among returnees. In fact, studies have demonstrated that reactions immediately following release from captivity or disaster are at times characterized by a sense of relief, euphoric mood and optimism [73, 74], followed by subsequent posttraumatic stress symptoms for some [75]. Additionally, since most symptoms were identified by clinicians using modified scales rather than through self-administered measures, there is a possibility that these reports did not fully capture the psychological state of the returnees. Alongside positive reactions, regressive behaviors were also observed, evident in increased clinginess of both children and adults, difficulty with decision making and assertiveness. The diverse reactions challenged staff’s expectations and called for flexible and adaptive responses in the immediate hours following their release from captivity. Furthermore, it highlighted the complexity of traumatic reactions, which are both highly individualistic and subject to a developmental trajectory that underlines the vitality of long-term follow-up [36].

Our findings also indicate that regressive reactions were evident among both children and adults. Although such responses are highly prevalent among traumatized children [76], regressive reactions are also evident among adult survivors of extreme trauma [27]. Similarly, facilitating social support, considered fundamental to effective child-focused interventions [77], is also crucial in treating traumatized adults [78]. Therefore, expertise in child trauma could benefit both adults and children, and inform the approach of professional teams. Hence, the decision to foster the care of returnees’ and their families during this highly vulnerable time, in the Children’s Hospital, and under the leadership of the child and adolescent hospital team, resulted in the creation of a flexible environment capable of meeting highly diverse needs across the developmental spectrum.

Efforts to obtain informative personal background *prior* to arrival of returnees was only partially effective. This required “learning on the go” as staff collected relevant information throughout returnees’ stay. This could be due to several factors. Firstly, the chaotic nature of the unprecedented terror attack, and the intense workload of state offices and agencies, could have hindered the efficient flow of returnees’ personal and medical background information to the hospital. Second, the names of hostages intended to be released, as well as the specific hospitals designated to receive them, were revealed only hours before their arrival, limiting the team’s ability to prepare and address multiple sources of information. Finally, as noted in previous mass disasters, such as the 9/11 terrorist attacks, considerable difficulties exist in creating synchronized organizational collaboration during disaster response, evidenced by insufficient information flow and inflexible hierarchies [79]. Thus, transmission of crucial background information in a timely manner was challenging.

Lastly, involving families in the preparation of returnee rooms was appreciated by family members and returnees, specifically the young children. Attention to individualized needs, as exemplified in bringing children’s pets to the hospital, proved to be helpful in the adjustment process. Implementation of a family-centered approach was effective and in line with recommendations pertaining to the care of pediatric patients, which has been previously found to enhance child and parental sense of control [80, 81]. Therefore, teams were keen on facilitating partnerships between medical staff and family members by involved family members in treatment planning, ensuring the presence of a parent or caregiver (for minors and adult returnees alike), and providing guidance on age- and developmentally-appropriate mitigation of events and their consequences.

### Optimal safety

The *Optimal Safety* component of the *ReSPOND* protocol proved greatly beneficial in guiding staff throughout their care of returnees. A consensus was reached and practiced with regards to the optimal environment to be maintained within the unit. Staff ensured returnee privacy at all times, attended to basic needs and followed recommended steps for enhancing returnees’ sense of self-efficacy with respect to age, developmental and familial roles. Yet a unique challenge was the interplay between individual psychological needs and the national-level needs associated with the larger hostage situation. Returnees were requested to participate in interviews conducted by secret service personnel. The purpose of these interviews was to swiftly obtain information that could shed light on the wellbeing of remaining hostages. Thus, hostages were faced with an extremely challenging task, while clinical staff were at times conflicted between the stated goal of reducing overstimulation versus the desire to assist returnees, as well as efforts to rescue others. Though minors under the age of 14 were excluded from interviews, clinical dilemmas pertaining to personnel allowed to accompany adolescents, the presence of a caregiver, the level of details requested and even the separation of adult caregivers from young children for the purpose of interviews, presented an array of challenges. Typically, these issues were resolved through professional multidisciplinary consultation and supervision, handled with sensitivity and the utmost transparency and empowerment.

### Navigating discharge

Determining optimal release and creating discharge plans were more challenging than anticipated. Initially, length of hospital stay was expected to be 24 to 48 hours, with a clear guideline towards returnees’ self-determination. The relatively brief duration that was initially recommended was based on studies emphasizing the importance of reintegrating hostages into their environments to reduce dependency on the medical system, a tendency often observed in survivors of captivity [82]. Furthermore, the need for medical clearance and the anticipated release of more hostages who would require the same designated facility, may have also contributed to the decision to limit the duration of hospitalization.

The evacuation and displacement of the majority of the returnees’ communities to temporary accommodations (i.e., hotels) resulted in additional challenges concerning the appropriate placement for continued care. While efforts were devoted to developing comprehensive bridging plans that connect returnees to ongoing medical, psychological, and social support services post release, follow-up regarding the effectiveness of these efforts was only partially obtained.

### Summary

The current paper discusses insights gained from developing and implementing the *Hostage-ReSPOND* protocol for receiving 29 Israeli civilians released from prolonged Hamas captivity. It emphasizes the rapid creation of a sensitive developmentally informed, multidisciplinary protocol for extreme trauma responses, proving effective despite challenges. A major strength of the protocol was related to the decision to foster the care of returnees and their families, during a highly vulnerable time, in the Children’s’ Hospital and under the leadership of the child and adolescent hospital team. This decision resulted in the creation of a flexible environment capable of meeting highly diverse needs across the developmental spectrum.

Another major strength was the pre-event training which recognized the diverse needs of returnees and successfully incorporated specifically tailored simulations pertinent to the care of released hostages across the age spectrum. This unique emphasis on training enabled teams to adapt to a novel protocol during an unprecedented and highly stressful hostage situation, resulting in an increased sense of professional competency. Moreover, specialized training helped healthcare teams navigate a highly complex situation, characterized by considerable diversity with regards to returnees’ ages, traumatic exposure, and personal circumstances, especially given the shared traumatic reality of providers as well following the October 7th terror attack.

Among the major challenges and limitations was the need for staff to operate within the context of an ongoing national mass trauma event, at times contributing to a sense of overwhelm. In addition, the displacement of the majority of the returnees to temporary lodgings due to large-scale evacuations, resulted in additional challenges concerning the appropriate placement for continued care, and limited the hospitals’ ability to maintain ongoing contact with returnees. Furthermore, the decision to limit the burden placed on returnees led to the implementation of proxy-clinician reports, which may have resulted in under-estimation of post-captivity symptoms. Finally, in several cases challenges arose regarding the division of labor, coordination of care, and collaboration, occasionally affecting the continuity and flow of care. Nevertheless, these were effectively addressed through consultations with senior clinical supervisors stationed at the ward. Overall, the work presented highlights the complexity of providing multidisciplinary care in extreme crisis situations, associated benefits, limitations and shortcomings, and the importance of evaluating and monitoring adaptive responses.

## Data Availability

All data presented can be requested from the authors.

## Abbreviations

MSR: Israel Center for Medical Simulation
PTSD: Post Traumatic Stress Disorder
ReSPOND: Readiness, Specialized teams, Optimal safety, and Navigating Discharge
I.M.P: Investigational Medicinal Product
IDF: Israel Defense Force
WHO: World Health Organization
SPIKES: Setting, Perception, Invitation, Knowledge and Empathy
TSCYC: Trauma Symptom Checklist for Young Children
TSCC: Trauma Symptom Checklist for Children
